# Epigenetic Regulation of Heart-ECHS∗

**DOI:** 10.1016/j.jacbts.2022.01.014

**Published:** 2022-04-25

**Authors:** Lu Gan, Liming Pei

**Affiliations:** a Center for Mitochondrial and Epigenomic Medicine, Department of Pathology and Laboratory Medicine, Children’s Hospital of Philadelphia, Philadelphia, Pennsylvania, USA; b Department of Pathology and Laboratory Medicine, Perelman School of Medicine, University of Pennsylvania, Philadelphia, Pennsylvania, USA; c Institute for Diabetes, Obesity, and Metabolism, Perelman School of Medicine, University of Pennsylvania, Philadelphia, Pennsylvania, USA; d Cardiovascular Institute, Perelman School of Medicine, University of Pennsylvania, Philadelphia, Pennsylvania, USA

**Keywords:** acetylation of H3K9, cardiomyopathy, enoyl-CoA hydratase 1, nicotinamide mononucleotide, p300

**Figure:**
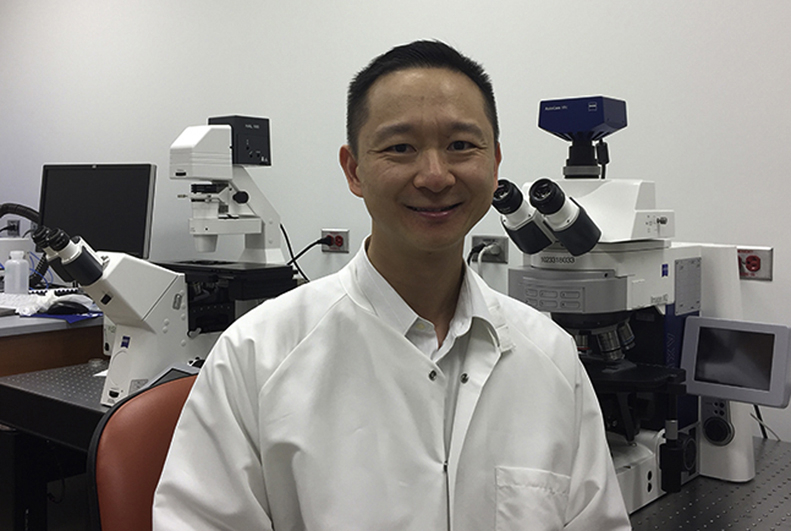

Cardiomyopathy represents a collection of diseases of the heart muscle that often leads to reduced cardiac function and even heart failure. Cardiomyopathy can be inherited or acquired and is estimated to affect up to 1 in 500 adults and people of all ages, races, and sexes. The constantly beating heart depends heavily on its highly abundant mitochondria to generate the energy to support its function. In addition, mitochondria-produced cellular metabolites, such as acetyl coenzyme A (acetyl-CoA) and S-adenosyl methionine, are essential substrates for multiple histone modification enzymes and can thereby affect the nuclear epigenome. These metabolites serve as key signals that sense cellular mitochondrial or metabolic status and translate it into nuclear gene expression changes.^1^ It is therefore not surprising that mutation of many genes important for mitochondrial energy production and metabolism can directly cause or contribute to both hereditary and acquired cardiomyopathy. Short-chain enoyl-CoA hydratase (ECHS1) is a key mitochondrial enzyme important for fatty acid (FA) β-oxidation (FAO) and branched-chain amino acid (BCAA) catabolism. Loss-of-function mutations of ECHS1 in humans cause a range of mild to severe symptoms that often include cardiomyopathy, but the underlying mechanisms are not well understood. In this issue of *JACC: Basic to Translational Science*, Cai et al^2^ report a new mechanism for ECHS1 in epigenetic regulation of cardiac fibroblast gene expression and cardiac fibrosis (Figure 1).

**Figure 1:**
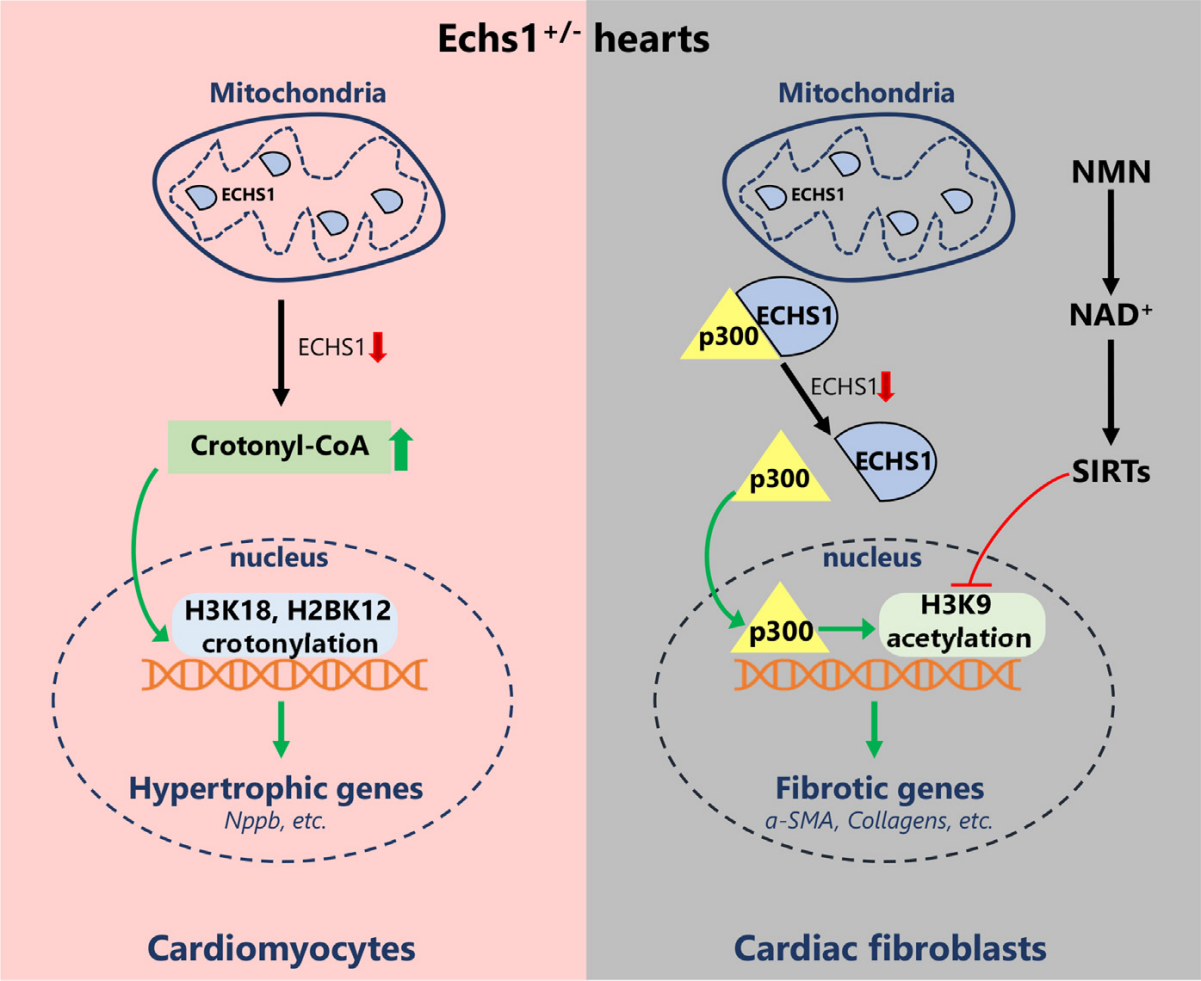
Cell Type–Specific Epigenetic Mechanisms for ECHS1 in Regulating Cardiac Gene Expression and Function In cardiomyocytes **(left)**, *Echs1* haploinsufficiency **(red arrow)** results in elevated crotonyl-CoA levels and increases the expression of hypertrophic genes via histone crotonylation of H3K18 and H2BK12. In cardiac fibroblasts **(right)**, *Echs1* haploinsufficiency promotes p300 nuclear translocation, leading to increased H3K9 acetylation and expression of fibrosis-related genes. Nicotinamide mononucleotide (NMN) diet ameliorates the cardiomyopathy and fibrosis phenotypes in *Echs1*^*+/−*^ mouse hearts, potentially by inhibiting H3K9 acetylation in cardiac fibroblasts. NAD^+^ = nicotinamide adenine dinucleotide; SIRT = sirtuin.

Posttranslational modifications of histones and other epigenetic mechanisms mediate dynamic regulation of gene transcription in various cardiac cell types and thus contribute to the development of many cardiovascular diseases. Targeting epigenetic processes is emerging as a potential therapeutic approach for cardiac diseases.^3^ In the mitochondrial FAO, the ECHS1 enzyme catalyzes the hydration of 2-trans-enoyl-CoA (such as crotonyl-CoA) intermediates to L-3-hydroxyacyl-CoA. It was recently discovered that ECHS1 deficiency led to elevated cellular crotonyl-CoA levels in cardiomyocytes, resulting in increased crotonylation of histone marks H3K18 and H2BK12.^4^ These epigenetic changes increased the cardiomyocyte expression of hypertrophic genes such as *Nppb* and contributed to Echs1-associated cardiomyopathy in mouse models. In the present study, Cai et al^2^ identified another new epigenetic mechanism of ECHS1 function in cardiac fibroblasts that modulates fibrotic gene expression and fibrosis.

Because *Echs1*-knockout mice exhibit embryonic lethality^5^, Cai et al^2^ carefully evaluated the cardiac functions of *Echs1*-heterozygous (*Echs1*^*+/−*^) mice in which the cardiac levels of both Echs1 RNA and protein are about one-half that of control. Both male and female *Echs1*^*+/−*^ mice showed decreased contractile function and increased heart weight, left ventricular internal dimension, and interventricular septum thickness, which were more pronounced in older mice. *Echs1*^*+/−*^ mice also presented diffuse myocardial fibrosis as early as 3 weeks of age, with increased α-smooth muscle actin, collagen I and collagen III RNA and protein levels in the heart. Consistent with the key function of ECHS1 in mitochondrial BCAA and FA metabolism, the authors observed increased cardiac BCAA and increased total FA levels in multiple tissues, including the hearts of *Echs1*^*+/−*^ mice. Intriguingly, the use of a diet with high FA and BCAA for wild-type (WT) mice induced similar increase of circulating and cardiac FA and BCAA as observed in *Echs1*^*+/−*^ mice on a chow diet. However, this dietary modification did not result in any cardiac dysfunction or fibrosis, suggesting potential mechanisms independent from FA and BCAA accumulation.

To investigate whether epigenetic mechanisms may explain these results, Cai et al^2^ examined multiple histone modifications. They found that H3K9 acetylation (H3K9Ac) was specifically increased in *Echs1*^*+/−*^ mouse hearts. Intriguingly, the H3K9Ac mark was previously linked to cardiac fibroblast activation and expression of fibrotic genes such as collagens.^6^ Here, the authors took a step further by looking at the cell-type specificity of this crucial epigenetic change. They found that the increase of H3K9Ac occurred only in primary cardiac fibroblasts from *Echs1*^*+/−*^ mice or in WT fibroblasts upon siRNA-mediated *Echs1* knockdown. In contrast, these changes were not observed in primary cardiomyocytes from *Echs1*^*+/−*^ mice or in WT cardiac myoblasts with *Echs1* knockdown. Furthermore, knockdown of other mitochondrial FAO enzymes did not alter H3K9Ac levels in either fibroblasts or cardiac myoblast cell lines, and *Echs1* knockdown resulted in decrease of acetyl-CoA only in mitochondria but not in the nucleus. These results led the authors to investigate other additional mechanisms underlying the cardiac fibroblast–specific H3K9Ac changes. Analyzing Echs1-interacting proteome results from their previous study,^7^ Cai et al^2^ found that Echs1 physically interacted with the histone acetyltransferase p300. While other known H3K9Ac-modifying enzymes remained unchanged in primary cardiac fibroblasts from *Echs1*^*+/−*^ mice, decreased Echs1 increased p300 translocation into the nucleus and consequently nuclear H3K9Ac levels. Notably, this was not observed in primary cardiomyocytes from *Echs1*^*+/−*^ mice.

As nicotinamide adenine dinucleotide (NAD^+^)–dependent histone deacetylase, some sirtuin proteins are known to reduce H3K9Ac levels. It was also previously reported that Sirt3 increased cellular Echs1 levels by inhibiting its degradation.^7^ Therefore, Cai et al^2^ tested whether adding nicotinamide mononucleotide (NMN), a precursor of NAD^+^, into the diet would alleviate the cardiomyopathy in *Echs1*^*+/−*^ mice. They found that the NMN diet increased cellular NAD^+^, Echs1, nuclear p300, and H3K9Ac levels in the *Echs1*^*+/−*^ mouse hearts. Importantly, the NMN diet resulted in mild but statistically significant improvement of cardiac function and largely prevented cardiac fibrosis in *Echs1*^*+/−*^ mice.

With new discoveries come new questions. Although ECHS1 interacts with p300 in both cardiomyocytes and cardiac fibroblasts, the inhibition of p300 nuclear translocation and ensuing H3K9Ac appear to occur only in fibroblasts. It remains little understood how this cell-type specificity is achieved. NMN diet is likely to affect many cellular NAD^+^-dependent pathways in multiple cardiac cell types. Which biological processes and cell types are most important for its benefit? Will NMN/NAD^+^ dietary supplementation helps patients with *ECHS1* mutations? The findings of Cai et al^2^ further expand the notion that mitochondrial proteins can regulate nuclear gene transcription through a variety of epigenetic mechanisms. In the case of *ECHS1*, it seems to engage distinct epigenetic mechanisms in a cell type–specific manner to modulate cardiac function and gene regulation (Figure 1). Future studies will undoubtedly discover additional mechanisms of mitochondria-to-nucleus retrograde signaling and elucidate their importance in both physiology and disease.

## Funding Support and Author Disclosures

Drs Gan and Pei are supported by the Office of the Assistant Secretary of Defense for Health Affairs through the Peer-Reviewed Medical Research Program under awards W81XWH20-1-0042, W81XWH20-1-0089, W81XWH22-1-0058, and NIH R01DK111495, U54HL156090. The authors have reported that they have no relationships relevant to the contents of this paper to disclose.
